# A Case of Multiple Polyps Causing Intussusception in an Adult Patient With Peutz-Jeghers Syndrome

**DOI:** 10.7759/cureus.30532

**Published:** 2022-10-20

**Authors:** Ebtehal S Alharbi, Jawaher S Alrumayh, Raneem H Alzaghran, Nada K Algaith, Abdel Nasser Shaheen

**Affiliations:** 1 Department of Surgical Oncology, College of Medicine, Qassim University, Qassim, SAU; 2 Department of Surgical Oncology, King Fahad Specialist Hospital, Qassim, SAU

**Keywords:** peutz-jeghers syndrome, bowel obstruction, emergency intervention, polyps, intussusception

## Abstract

Despite intussusception being less prevalent among adults, its effects are severe and often require emergency intervention. Peutz-Jeghers syndrome is a rare autosomal dominant syndrome that leads to the growth of polyps in the gastrointestinal mucosa. In this case report, we present the case of a 26-year-old man who was brought to the emergency room complaining of crampy abdominal pain, vomiting, and constipation. Intussusception was observed on imaging and confirmed at surgery. The necrotic parts of the small bowel were resected. Postoperatively, the patient was stable, had minimum pain, and did not have any complications throughout the hospital stay. He was discharged home on day seven and advised to follow up. The course at the one-month follow-up was uneventful with no similar episodes. This case report is intended as a reminder for emergency physicians to consider intussusception as a potential diagnosis in patients presenting with abdominal pain and bowel obstruction because the symptoms are often non-specific and episodic.

## Introduction

Peutz-Jeghers syndrome (PJS) is a rare autosomal dominant disorder characterized by hyperpigmented mucocutaneous macules and the growth of non-cancerous hamartomatous polyps in the gastrointestinal tract, as well as an increased risk of malignancy [1]. PJS increases the susceptibility to intussusception. Intussusception is common in children aged between six and 18 months but rare among adults [2]. There is a significant difference between pediatric and adult intussusception in terms of the etiology, clinical features, and treatment [3]. Polyps in the gastrointestinal tract are a known cause of intussusception, especially when they enlarge and cause intestinal obstruction [1].

We report intussusception in a 26-year-old male patient who was known to have PJS. Unfortunately, the diagnosis was not made until he subsequently developed bowel obstruction due to intussusception, necessitating exploratory bowel resection. This case report highlights the importance of early diagnosis, close monitoring, and timely intervention in PJS-related intussusception.

## Case presentation

A 26-year-old man with clinically diagnosed PJS since childhood presented to the emergency department twice within three days complaining of intermittent crampy abdominal pain, vomiting, and constipation for six days and melena for 10 days. He had a history of inguinal hernia repair two years prior. His family history was unknown and he had not experienced pain of this severity previously. The other systems were unremarkable.

Abdominal examination revealed mild distension, diffuse tenderness, and a fusiform bulge in the mid-to-lower abdomen. The rectum was devoid of stool and masses. There were hyperpigmented spots on the face, the palm of the hands, and mucous membranes.

The patient on the first arrival was hemodynamically stable with a blood pressure of 119/74 mmHg, a pulse of 122 beats/minute, afebrile, and oxygen saturation of 95% on room air. His laboratory results demonstrated anemia (hemoglobin level of 10.4 g/dL) and hypokalemia (2.8 mEq/L). The remaining results were unremarkable.

**Figure 1:**
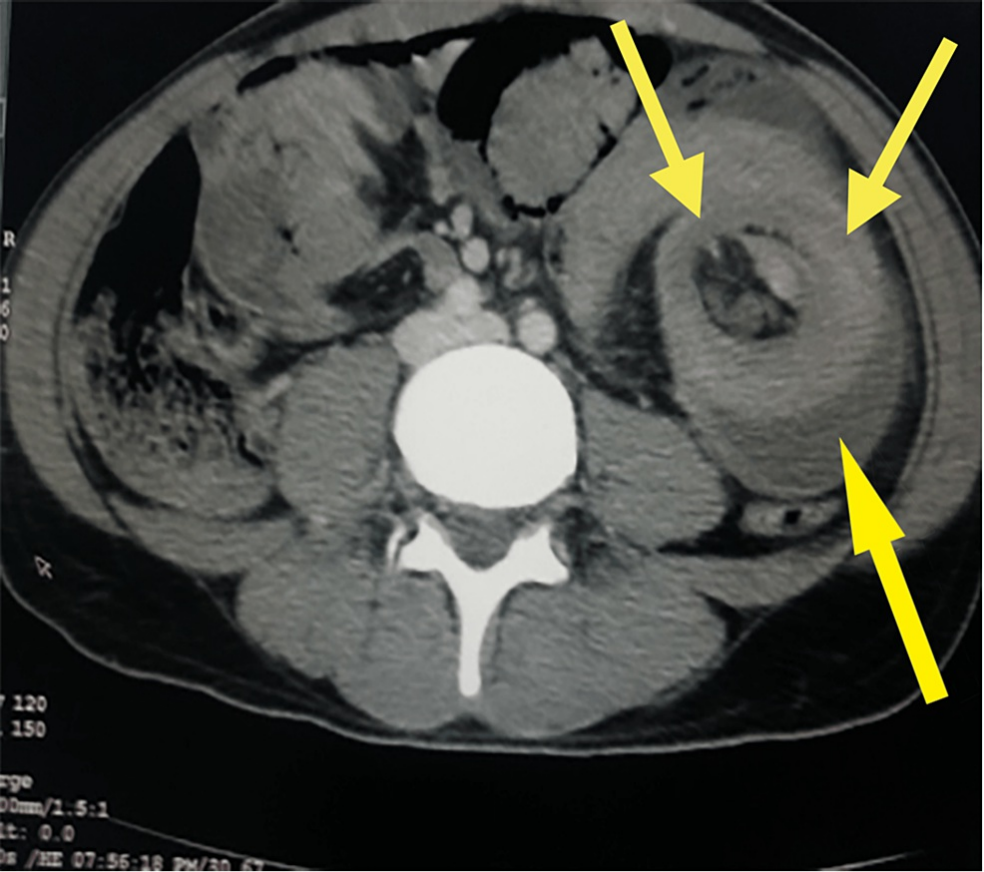
Preoperative computed tomography showing the target sign of intussusception (yellow arrow) which indicates intestinal obstruction. The obstruction was caused by intussusception 2 m from the duodenojejunal junction.

Multiple fluid levels were observed on erect abdominal radiography. Computed tomography (CT) of the abdomen showed a target sign which indicates intestinal obstruction (Figure 1). The radiographs indicated that it could either be volvulus or intussusception. Therefore, he underwent an emergency laparotomy.

**Figure 2:**
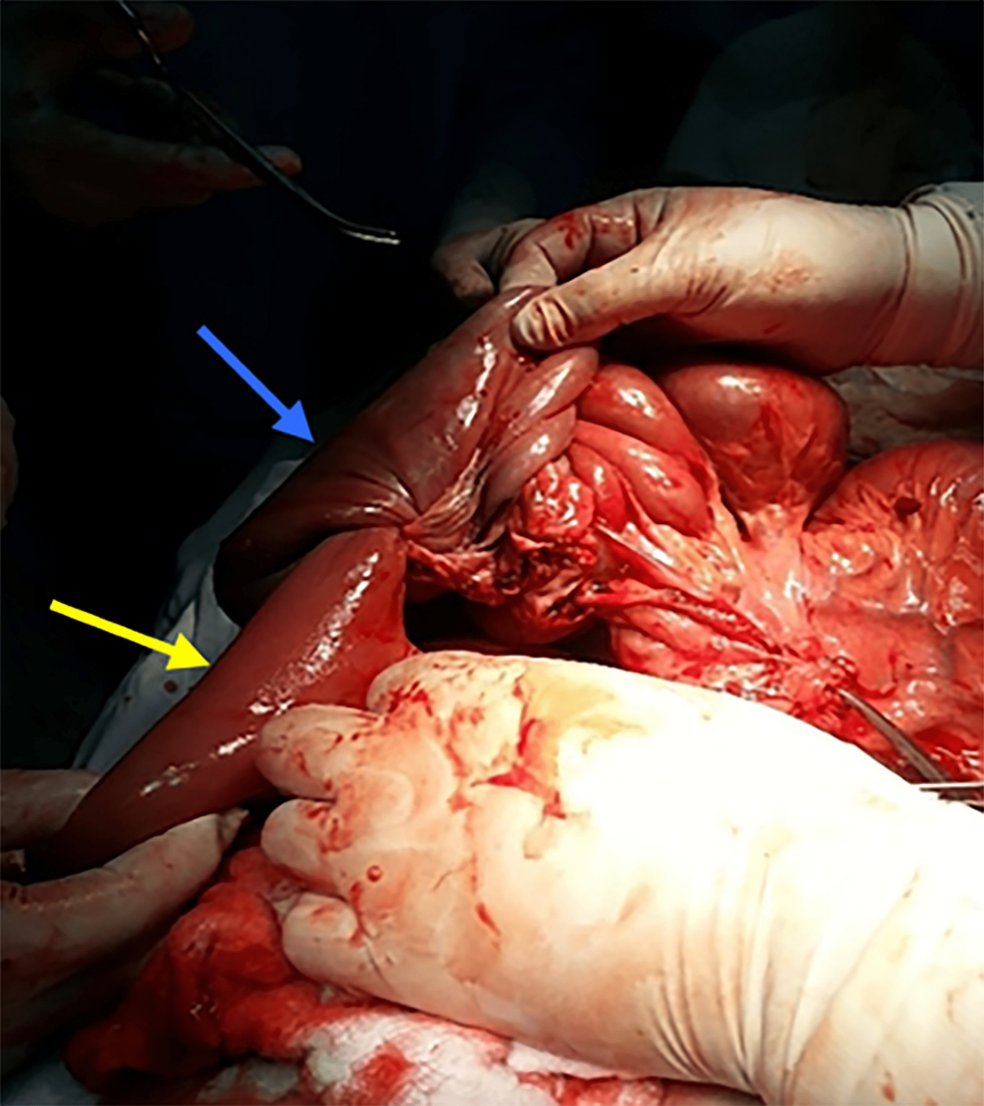
Intraoperative photograph of the small bowel transition point where the intussusception was found (yellow arrow: intussusception, blue arrow: intussuscipiens).

**Figure 3:**
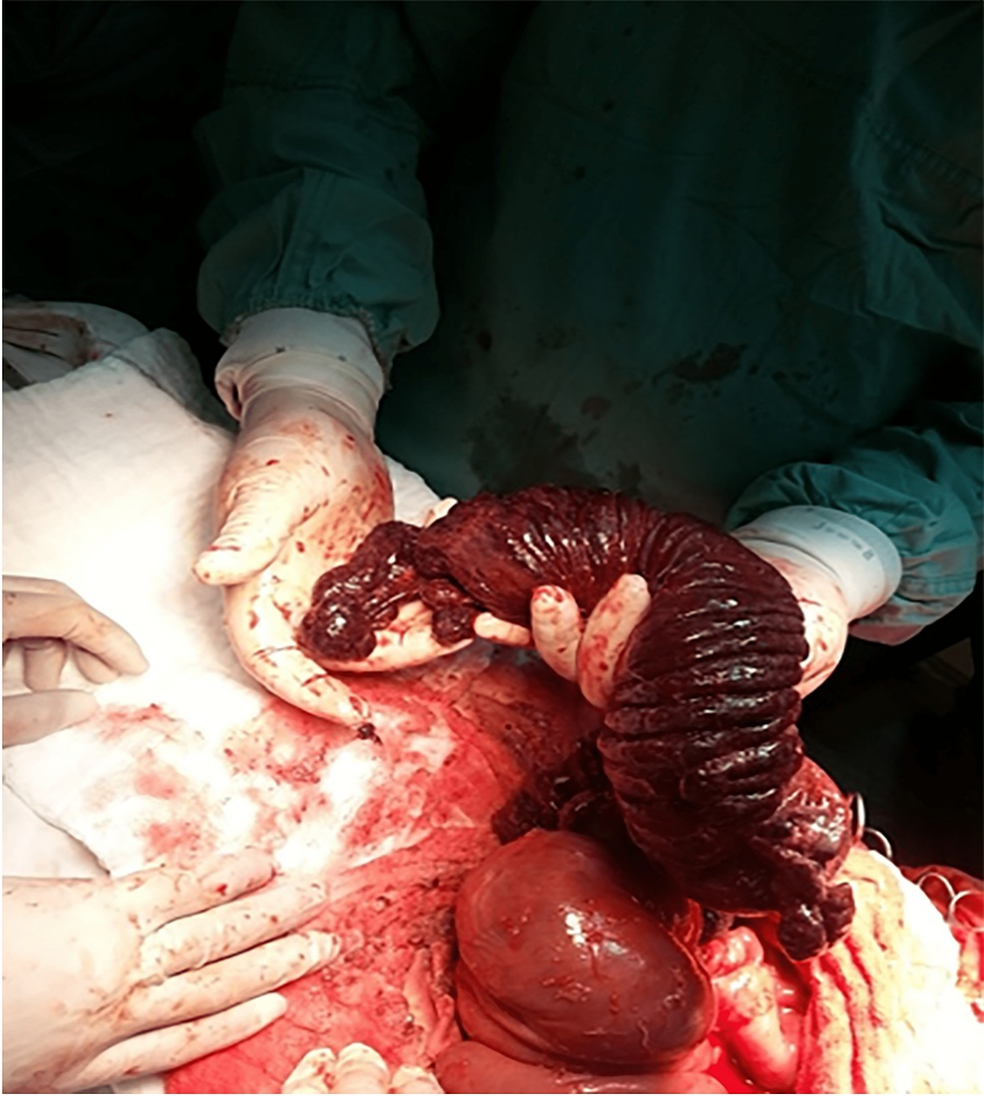
Intraoperative photograph of the infarction of the small intestine (dilated bowel) due to the obstruction. This section was resected and sent for pathological examination.

The bowel was evaluated, and the presence of an intestinal obstruction caused by intussusception was identified 2 m away from the duodenojejunal junction. The small bowel transition point, intussusception, and intussuscipiens are shown in Figure 2. Multiple polyps of varying sizes were found intraluminally. The infarcted segment of the small intestine, which was 1.5 m long, was resected and sent for pathological examination (Figure 3). The telescoped portions of the small bowel and the necrotic tissue were resected and anastomosis was performed.

The pathology report showed the presence of hemorrhagic infarction with focally thrombosed blood vessels and multiple hamartomatous polyps ranging in size from a few mm to 5 cm with marked necrosis. The margins of the specimen were congested and edematous, and the mucosa was necrotic and hemorrhagic. Lymph nodes were reactive with no dysplasia.

Postoperatively, our patient was stable, had minimum pain, did not have any complications throughout the hospital stay, and passed the postoperative course smoothly. He was discharged home on day seven and advised to follow up. The course at the one-month follow-up was uneventful with no similar episodes.

## Discussion

PJS is a rare hereditary condition of autosomal dominant inheritance, with a prevalence of 1 in 10,000 patients [4]. It is characterized by hamartomatous polyps within the gastrointestinal tract and increased susceptibility to malignancy [1]. PJS can arise from spontaneous mutations, though it is most often associated with autosomal dominant inheritance. Mutations involving the STK11 tumor-suppressor gene, located on chromosome 19p13.3, can also cause PJS [5].

According to the World Health Organization, three categories of patients should be frequently screened for PJS. Those with a family history of PJS, individuals who exhibit histologically three or more Peutz-Jeghers polyps, and those with a family history of mucocutaneous pigmentation [6]. The most common visible clinical feature of PJS is pigmentation, which often occurs in the buccal mucosa and the lips. The polyps associated with PJS often manifest during teenage years and early adulthood [7]. It has been widely observed that 90% of patients with PJS will develop small intestinal polyps [3]. PJS is prevalent in both sexes almost equally [1].

The most common clinical manifestations of PJS include abdominal pain, occult bleeding, diarrhea, vomiting, and intestinal obstruction due to intussusception which is caused by polyps [8]. Previous studies have indicated that patients with PJS have a higher risk of gastrointestinal malignancies compared to others. In a meta-analysis of six studies, patients aged between 15 and 64 years had a malignancy risk greater than 90% [9]. Patients with confirmed PJS require frequent surveillance on a regular basis to avoid complications and enhance their prognosis [10].

Despite intussusception being rare among adults, it is often a medical emergency. Intussusception is more common in infants and children aged between six months and two years [11]. The incidence in adults is about 0.02% of all hospital admissions with over 80% secondary to a focal point. Benign, malignant, or idiopathic lesions could be the lead point that caused intussusceptions [12].

In patients with PJS, intussusception is diagnosed by a history of the syndrome and physical examination. The most common findings are abdominal distention and pain, which were present in our case [13].

For our patient, reduction was not feasible. We performed a laparotomy due to intestinal obstruction which revealed further intussusception with a necrotic small bowel segment that required resection and anastomosis.

## Conclusions

Intussusception can be caused by PJS polyps. In adults, it is an infrequent problem and is difficult to diagnose because the symptoms are often non-specific and episodic. Thus, it might be easily misdiagnosed in the emergency department. Bowel screening is essential in patients with PJS because it aids in the early detection of polyps and helps avoid the need for urgent surgery to treat intestinal intussusception. Thus, in the case of intussusception, which is uncommon in adults, it is crucial to carefully review the patient’s medical history, physical examination findings, and radiological and endoscopic results.
